# A Comparative Study on the Quality of MRI Reporting in Primary Rectal Cancer

**DOI:** 10.7759/cureus.48730

**Published:** 2023-11-13

**Authors:** Basil Ibrahim, Ahmmad Alfatih, Ammara Shafiq, Mohammed Arifuzaman

**Affiliations:** 1 Surgery, Manchester Foundation Trust, Manchester, GBR; 2 Vascular Surgery, University Hospital Galway, Galway, IRL

**Keywords:** standardized template, preoperative imaging, quality of reporting, rectal cancer, mri pelvis

## Abstract

Background

Rectal cancer is a widespread health concern in the UK, and MRI is vital for accurate diagnosis and effective treatment. To enhance the quality of MRI reports for rectal cancer, structured reporting can be utilized. Despite the existence of global guidelines and templates, there is a lack of use for standardized templates. Thus, this study seeks to assess and compare the quality of MRI reports in free text style for rectal cancer at a single hospital with international recommendations.

Methodology

We conducted a retrospective cohort study on adult patients diagnosed with primary rectal cancer and underwent an MRI of the rectum/pelvis. The study aimed to identify and compare the difference in reporting quality between in-house GI radiologists and out-of-hours outsourced reporting agencies. The quality of reporting was identified based on at least fifteen selected features recommended by the European Society of Gastrointestinal and Abdominal Radiology and the Society of Abdominal Radiology. The study was performed in a General and Colorectal Surgery Department in the North East of England.

Results

The study retrospectively analysed 94 reports of primary rectal cancer patients over three years. The quality of reporting was compared between in-house GI radiologists and out-of-hours outsourced reporting agencies. The results showed that in-house radiologists had better reporting quality than outsourced agencies in terms of TNM stage (TNM is a notation system that describes the stage of cancer), predicted extramural venous invasion (EMVI), mesorectal fascia involvement (MRF), T stage, and shape. No statistical significance was found for metastasis, node status, further MRF description, peritoneal reflection, or MRI signal.

Conclusion

The study found that local GI radiologists had better quality reporting than outsourced agencies for rectal cancer MRI reports, but still missed important features. A unified structured reporting template is recommended to improve the quality of MRI reporting for rectal cancer.

## Introduction

Colorectal cancer is the third most common cancer among men and women in the United Kingdom after prostate/breast and lung respectively [1]. There are around 42,900 new bowel cancer cases in the UK every year, that's nearly 120 every day (2016-2018) with the rectum being the most common specific location for bowel cancers. This type of cancer is more common in developed countries compared to less developed ones. Nonetheless, the death rate in developed nations is lower due to improved screening methods and better treatment options for rectal cancer [2].

With the current paradigm shift towards neoadjuvant therapy improving patient outcomes, tumour staging using magnetic resonance imaging (MRI) has become the essential modality in the diagnosis and management of rectal cancer. MRI can precisely describe the tumour location, lymphovascular invasion, nodal status, and extra-mural invasion (EMVI), guiding towards the best available treatment option; neoadjuvant chemo/radiotherapy (CRT) and potential surgical planning [3]. MRI can be helpful in various ways for patients with locally advanced rectal cancer (LARC) before surgery. It can identify patients who are suitable for neoadjuvant CRT treatment and assist surgeons in their surgical planning. Additionally, it can identify poor prognostic factors such as extramural vascular invasion (EMVI), mucin content, and involvement of the mesorectal fascia (MRF).

MRI assessment of tumour regression grade (TRG) and circumferential resection margin (CRM) can also predict survival outcomes for both good and poor responders. This allows the multidisciplinary team to offer additional treatment options before planning definitive surgery. The potential benefits achieved with rectal MRI are strictly dependent on obtaining good-quality images to allow for the characterization of the main anatomic structures and their relation to the tumour [4]. High-spatial-resolution T2-weighted imaging is the most important MRI sequence in the evaluation of rectal cancer and anatomic structures. Standardized imaging protocols also allow for more accurate and reproducible interpretations, which facilitate the widespread use of this technique [5].

Quality reporting of the MRI is crucial for the accurate transfer of information among different specialties and hence a systematic, structured approach is recommended. Numerous studies have depicted that structured reporting via a pre-designed template improves the quality of MRI performed for rectal cancer in contrast to free text formats [6,7]. Template reporting ensures that key data points like EMVI, circumferential resection margin (CRM), and nodal status are recorded routinely. In this regard, several guidelines and reporting templates have been proposed and adopted globally [8-10]. Beets-Tan et al. proposed a template for the MRI reporting of rectal cancer based on the recommendations of the European Society of Gastrointestinal and Abdominal Radiology (ESGAR) consensus meeting in 2016 [10]. The template is suggested for both primary staging and restaging followed by neoadjuvant therapy and has been unanimously recommended by the panel of ESGAR. Similarly, the Korean Society of Abdominal Radiology enlists the essential items for the structured reporting of MRI rectal cancer; tumour length, circumferential margin, extramural tumour invasion, shortest tumour distance from the mesorectal fascia, T staging, nodal spread (mesorectal and extra mesorectal), anal canal involvement and EMV [9]. German Radiological Society has also published a template for MRI rectal cancer which entails all significant information about the rectal tumour [11]. Standardized reporting not only improves the quality of radiology reports but also makes them reproducible, and a similar MRI rectal staging protocol has also been recommended by the French Radiology Group (GRERCAR) and the Surgical Group for rectal cancer [3].

The MRI reporting templates for rectal cancer have been adopted in many countries all around the world. However, we were unable to find an established template from the radiologists with a consensus agreement that is used nationally in the United Kingdom. This study aims to assess and compare the quality of MRI reports in a single District General Hospital for rectal cancers with SAR and ESGAR recommendations [10,12]. The secondary aim was to identify any differences in the quality of reporting between in-house gastrointestinal radiologists and out-of-hours resources.

## Materials and methods

We performed a retrospective cohort study guided by the Strengthening the Reporting of Cohort Studies in Surgery (STROCSS) guidelines for observational studies [13]. As we utilized non-identifiable hospital data and the study was retrospective, we deemed it unnecessary to obtain approval from a Research Ethics Committee or patient consent. The study was undertaken in a General and Colorectal Surgery Department in the North East region of England at the University Hospital of North Durham. All adult patients over the age of 18 who have been diagnosed with primary rectal cancer and underwent MRI rectum/pelvis over a period of 3 years between 2018 and 2021 were identified using the medical records and included in this study. Any patients with re-staging or post-treatment MRI rectum/pelvis were excluded. 

We aimed to assess the quality of MRI reporting using the free text style usually performed by radiologists as well as to identify and compare the differences in reporting quality between the in-house GI radiologist and out-of-hours from outsourced reporting agencies. The quality of reporting was identified if at least 15 features were selected in concordance with the updated recommendations of the European Society of Gastrointestinal and Abdominal Radiology and the Society of Abdominal Radiology [10,12]. An added quality feature of whether the local GI radiologist made the report, or an out-of-hours private reporting facility was considered and documented in our analysis.

The data was gathered through the utilization of a comprehensive electronic data collection form, which included the following parameters: age, gender, vertical location, length, distance from the anal verge, radial location, shape, MRI signal, relationship to the peritoneal reflection, T-stage, detailed measurements in the event that the stage exceeded T3, MRF, further MRF description if threatened or involved, EMVI, node status, metastasis, TNM statement, and in-house GI radiologist or out-of-hours external radiologist.

An initial overall frequency analysis was performed on the highlighted variables. Following this, a comparison was made between the in-house GI radiologist reporting style and the out-of-hours reporting style. This allowed for a comprehensive evaluation of the differences and similarities in the way information was conveyed and presented by these two. In order to perform a multiple regression analysis, the researchers utilized the Stats Direct 3 software (StatsDirect Ltd, Wirral, UK).

## Results

A total number of 94 patients who had MRIs for primary rectal cancer were identified and selected retrospectively from our local radiology system covering three years. A number of 86 reports were retrieved from the local radiology system and cross-checked with our selection criteria of only primary rectal cancer excluding re-staging and post-treatment reports.

Of the 86 patients’ reports covering a three-year duration, 56% were males and 44% were females with a median age of 66.5 years. An initial overall frequency analysis of the highlighted variables was performed followed by a comparison of both in-house and out-of-hours reporting styles (Tables 1, 2).

**Table 1:** An overall assessment of the frequency of identified features mentioned in all reports TNM: The most widely used cancer staging system; MRF: Mesorectal fascia; EMVI: Extramural vascular invasion

	"YES" Count	Frequency (Rounded up)
Vertical Location	84	98%
Length	78	91%
Distance from Anal Verge	67	78%
Radial Location	45	52%
Shape	31	36%
MRI Signal	9	10%
Relationship to Peritoneal Reflection	30	35%
T Stage	81	94%
MRF	75	87%
EMVI	57	66%
Nodes Status	82	95%
TNM Statement	79	92%

**Table 2:** A comparison between in-house GI radiologist and out-of-hours/private radiologist reports assessing the frequency of commenting on relevant features TNM: The most widely used cancer staging system; MRF: Mesorectal fascia; EMVI: Extramural vascular invasion

Category	In-house	In-house Freq.	Out-of-hours	Out-of-hours Freq.
Vertical Location	75	97%	9	100%
Length	71	92%	7	78%
Distance from Anal Verge	60	78%	7	78%
Radial Location	40	52%	5	56%
Shape	25	32%	6	67%
MRI Signal	7	9%	2	22%
Relationship to Peritoneal Reflection	29	38%	1	11%
T Stage	74	96%	7	78%
MRF	69	90%	6	67%
EMVI	54	70%	3	33%
Nodes Status	74	96%	8	89%
TNM Statement	73	95%	6	67%

The best quality was observed in features such as vertical location (98%), length (91%), T stage (94%), nodes status (95%), and TNM statement (92%). Other important features were not commented on as much in the reports like mesorectal fascia (MRF) status (87%), distance from the anal verge (78%), and extramural vascular invasion (EMVI) (66%). On the other hand, there were no satisfactory results with the rest of the measured descriptors.

**Figure 1:**
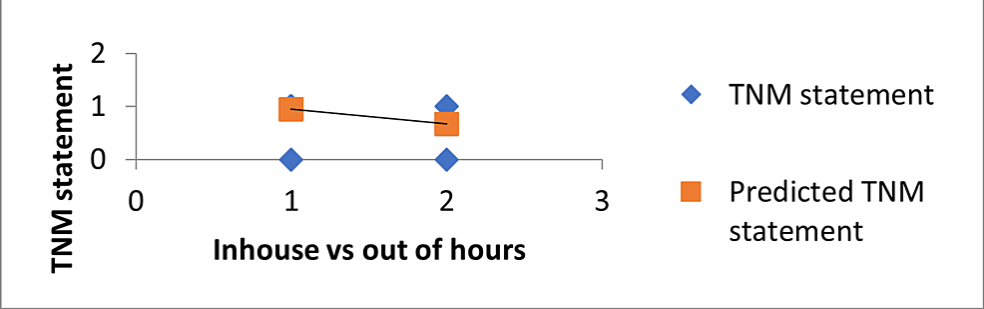
A comparison of TNM statements reported by in-house and out-of-hours radiologists The image shows that the in-house radiologist reporting had significantly better TNM statements when compared to the out-of-hours radiologist. TNM: The most widely used cancer staging system

Multiple regression analysis was conducted using the Stats Direct 3 software with a confidence interval of 95%. The in-house GI radiologist reporting was better with statistically significant reporting for the TNM stage (p = 0.003) (Table 2 and Figure 1), predicted EMVI (p = 0.027), MRF (p = 0.051), T stage (p = 0.026), shape (p = 0.043). There was no statistical significance associated with metastasis, node status, further MRF description if threatened or involved, peritoneal reflection, or MRI signal. None of the statistical results showed better results with out-of-hours reporting when compared to in-house GI radiologists.

## Discussion

We conducted a retrospective cohort study to assess the free text reporting style for the MRI rectum in primary rectal cancers as well as to compare the quality of reporting between in-house gastrointestinal radiologists and out-of-hours resources. Overall, there was a lack of detailed reporting across all reporters with evidence of missing important features. There was also a significant difference in reporting quality between gastrointestinal radiologists and outsourced radiologists.

A consensus agreement on a nationally used MRI reporting template for rectal cancer remains elusive in the United Kingdom despite its widespread adoption in many countries worldwide. The absence of a standardized template poses a significant challenge to the effective management of rectal cancer, potentially leading to inconsistencies in the interpretation of radiological findings and the development of treatment plans. This underscores the need for concerted efforts by relevant bodies in the UK to work collaboratively towards formulating a consensus-based MRI reporting template that is comprehensive, accurate, and meets the needs of all professionals involved in the diagnosis and management of rectal cancer.

Gupta et al. conducted a study to determine how using a specific template for reporting rectal cancer via MRI impacted a hospital [7]. The researchers created a template that contained 14 necessary parameters to be included in the reports. They retrospectively analyzed 100 rectal MRI reports, with 50 reports created before the template was used and 50 reports created after its implementation. They checked each report to see if the 14 parameters were present. The data revealed that the template significantly improved the number of parameters reported. The total number of parameters reported increased from 10 to 14. Prior to the template's introduction, some parameters were frequently missing, such as T staging, restricted diffusion, anterior peritoneal reflection (APR) involvement, and extramural vascular invasion. However, after the template's implementation, the reporting of these parameters improved to 98-100%. The most significant improvements were seen in T staging, restricted diffusion on DWI, and APR involvement. These parameters went from being unreported in a low percentage of cases to being reported in almost all cases. In addition to analyzing the reports, the researchers conducted an anonymous online feedback survey with members of the colorectal tumour board after the template's introduction. The survey showed that 100% of respondents felt less need to speak with the radiologist to clarify the report, 81.8% felt an improvement in the report's quality compared to free-style reports, and 91% felt that the new template was easier to interpret. The template increased the number of necessary imaging parameters mentioned in the reports, reducing the need for clarification, and improving the overall quality of reporting [7].

A team of 20 abdominal imaging experts from the Society of Abdominal Radiology (SAR) aimed to improve structured MRI reports for the primary staging of rectal cancer by creating a new report based on expert opinions and a review of relevant literature. To do this, they completed a questionnaire consisting of 22 items related to rectal cancer staging that were classified as "appropriate," "inappropriate," or "needs group discussion" based on consensus percentages. Sixteen items required further discussion and consensus, and after group discussion, consensus was achieved for 21 of the items. The consensus meeting led to the development of a revised structured report for MRI staging of rectal cancer, which included significant modifications such as excluding the T2/early T3 category, replacing "circumferential resection margin (CRM)" with "mesorectal fascia (MRF)," revising the definition of "mucinous content," creating two categories for suspicious lymph nodes (LNs) and tumour deposits, and classifying suspicious extra-mesorectal lymph nodes (LNs) by anatomic location. The authors recommend using this new structured reporting template for primary MRI staging of rectal cancer to improve standardized reporting, consistency, and clarity in communicating MRI findings for rectal cancer staging. The SAR Disease Focused Panel on Rectal and Anal Cancer developed this updated reporting template based on expert consensus and a review of the literature, intending to enhance accuracy and communication in the management of rectal cancer patients [14].

Another example was a study conducted to evaluate the impact of a two-day intensive pelvic MRI workshop on an experienced radiologist's reporting skills. The study involved 42 patients, and a 30-point MRI proforma was used to score the pre-treatment high-resolution MRI scans. The radiologist evaluated the scans initially, followed by a re-evaluation immediately after the workshop, and then another re-evaluation after one year. The radiologist was unaware of previous reports, their performance, or the pathology. The reports generated by the radiologist were compared to those by an expert radiologist on each occasion, and inter-observer agreement was measured using Cohen's kappa. After the workshop training, there was a remarkable improvement in the mean tumour height and distance from the anal verge, reduced to just 1 mm. The nodal positivity also demonstrated a significant improvement over time, starting with fair agreement, followed by moderate agreement after the workshop, and eventually good agreement after a year. The trend was similar for detecting extramural vascular invasion (EMVI) positivity and circumferential resection margin (CRM) positivity. The detection of advanced tumours also improved, while the detection of early-stage disease showed the highest agreement after one year [15].

It is important to note that our study has some potential limitations and biases. Firstly, the study design was retrospective, which means that it is subject to inherent limitations that should be taken into account when interpreting the findings. For instance, it was not possible to clarify how much time was spent by each reporter. Secondly, we were unable to identify whether the radiologists from the out-of-hours reporting agency were GI radiologists or not, due to the retrospective nature of the study. Lastly, the study was conducted at a single center and may not be the best representative of the entire population.

## Conclusions

This study assessed and compared the quality of MRI reports in free text style for rectal cancer at a single hospital. Based on our results, we conclude there was a significant difference in the quality of MRI reporting in primary rectal cancer in favour of local gastrointestinal radiologists; however, they have also missed important features required for a complete assessment of rectal cancers. We propose that colorectal departments should have a unified structured reporting template, and this should be adopted by hospitals with such a service.
